# Causal effects of gut microbiota on diabetic retinopathy: A Mendelian randomization study

**DOI:** 10.3389/fimmu.2022.930318

**Published:** 2022-09-08

**Authors:** Kangcheng Liu, Jing Zou, Huimin Fan, Hanying Hu, Zhipeng You

**Affiliations:** ^1^ Jiangxi Province Division of National Clinical Research Center for Ocular Diseases, Jiangxi Clinical Research Center for Ophthalmic Disease, Jiangxi Research Institute of Ophthalmology and Visual Science, Affiliated Eye Hospital of Nanchang University, Nanchang, China; ^2^ Hunan Key Laboratory of Ophthalmology, Eye Center of Xiangya Hospital, Central South University, Changsha, China

**Keywords:** diabetic retinopathy, gut microbiota, Mendelian randomization, causality, gut-retina axis

## Abstract

**Background:**

Previous researches have implicated a vital association between gut microbiota (GM) and diabetic retinopathy (DR) based on the association of the “gut-retina” axis. But their causal relationship has not been elucidated.

**Methods:**

Instrumental variables of 211 GM taxa were obtained from genome wide association study (GWAS), and Mendelian randomization study was carried out to estimate their effects on DR risk from FinnGen GWAS (14,584 DR cases and 202,082 controls). Inverse variance weighted (IVW) is the main method to analyze causality, and MR results are verified by several sensitive analyses.

**Results:**

As for 211 GM taxa, IVW results confirmed that family-*Christensenellaceae* (*P* = 1.36×10^-2^) and family-*Peptococcaceae* (*P* = 3.13×10^-2^) were protective factors for DR. Genus-*Ruminococcaceae_UCG_011* (*P* = 4.83×10^-3^), genus-*Eubacterium_rectale_group* (*P* = 3.44×10^-2^) and genus-*Adlercreutzia* (*P* = 4.82×10^-2^) were correlated with the risk of DR. At the phylum, class and order levels, we found no GM taxa that were causally related to DR (*P*>0.05). Heterogeneity (*P*>0.05) and pleiotropy (*P*>0.05) analysis confirmed the robustness of MR results.

**Conclusion:**

We confirmed that there was a potential causal relationship between some GM taxa and DR, which highlights the association of the “gut-retina” axis and offered new insights into the GM-mediated mechanism of DR. Further explorations of their association are required and will lead to find new biomarkers for targeted prevention strategies of DR.

## 1 Introduction

Diabetes is one of the metabolic disorders with increasing incidence and is predicted to globally affect more than 578 million individuals by 2030 (1). As a microvascular diabetic complication, diabetic retinopathy (DR) is one of the most prevalent metabolic and blinding eye diseases in ophthalmology (2). Current research indicates that the complex mechanism of DR involves the interaction of multiple factors, including genetics, angiogenesis, immunity, inflammatory injury, and neurodegeneration (3). Therefore, researchers have proposed and developed more targeted treatment and prevention approaches. Based on these mechanisms, DR presents a significant burden on global health, and the current therapeutic strategies are unsatisfactory. Interestingly, not all diabetic patients develop DR, and underlying mechanisms remain unexplained. Besides, whether there exist other factors than the documented metabolic factors contributing to individual differences remains unclear.

Individual differences could be attributed to changes in the dynamic balance of gut microbiota (GM) in the human body (4). Reports indicate that the ecological dysregulation of GM in the human body could trigger the development of inflammatory, metabolic, mental, and immune diseases (5–8). Studies have reported a relationship between GM and eye diseases (9). Therefore, whether GM is likely to affect individuals with DR remains elusive. Rowan et al. (10) proposed the concept of a “gut-retina” axis and revealed that GM modulates retinal disease. Prasad et al. (11) assessed plasma, retina, and fecal from diabetic mice and discovered that intestinal flora promotes damage and inflammation in the retina. Beli et al. (12) disrupted the ecological profile of GM in mice by intermittent fasting (IF) and discovered that it exhibited a preventive effect against DR and prolonged their survival period. In a cross-sectional study of patients with DR, Khan et al. (13) noted that the relative abundance ratio of *Bacteroidetes* to *Firmicutes* was linked to DR. However, Khan did not identify differences in the abundance of specific GM taxa in DR patients compared to the controls.

Meanwhile, research on the mechanisms of the “gut-retina” axis in DR patients has been significantly suppressed by various GM. The human gastrointestinal tract is affected by multiple factors, including diet and rest. Therefore human studies that have established the relationship between specific GM and DR are inadequate.

In contrast to randomized controlled studies, Mendelian randomization (MR) studies prevent the possible effects of confounding factors (e.g., diet) and reverse causality, using genetic variation instead of exposure to assess causal associations with outcomes. Studies using MR analysis to explore the causal relationship between GM and autoimmune diseases (14) and neuropsychiatric conditions have matured (15). Nonetheless, the mechanisms by which DR applies MR analysis remain unexplored.

This work selected GM taxa as exposure and DR as an outcome for MR analysis to explore the causal relationship and provide a theoretical basis for further research into the complex DR mechanisms. Moreover, novel biomarkers and diagnostic and therapeutic strategies could be provided by identifying the relationship between specific GM and DR patients.

## 2 Materials and methods

### 2.1 The assumptions and study design of MR

**Figure 1:**
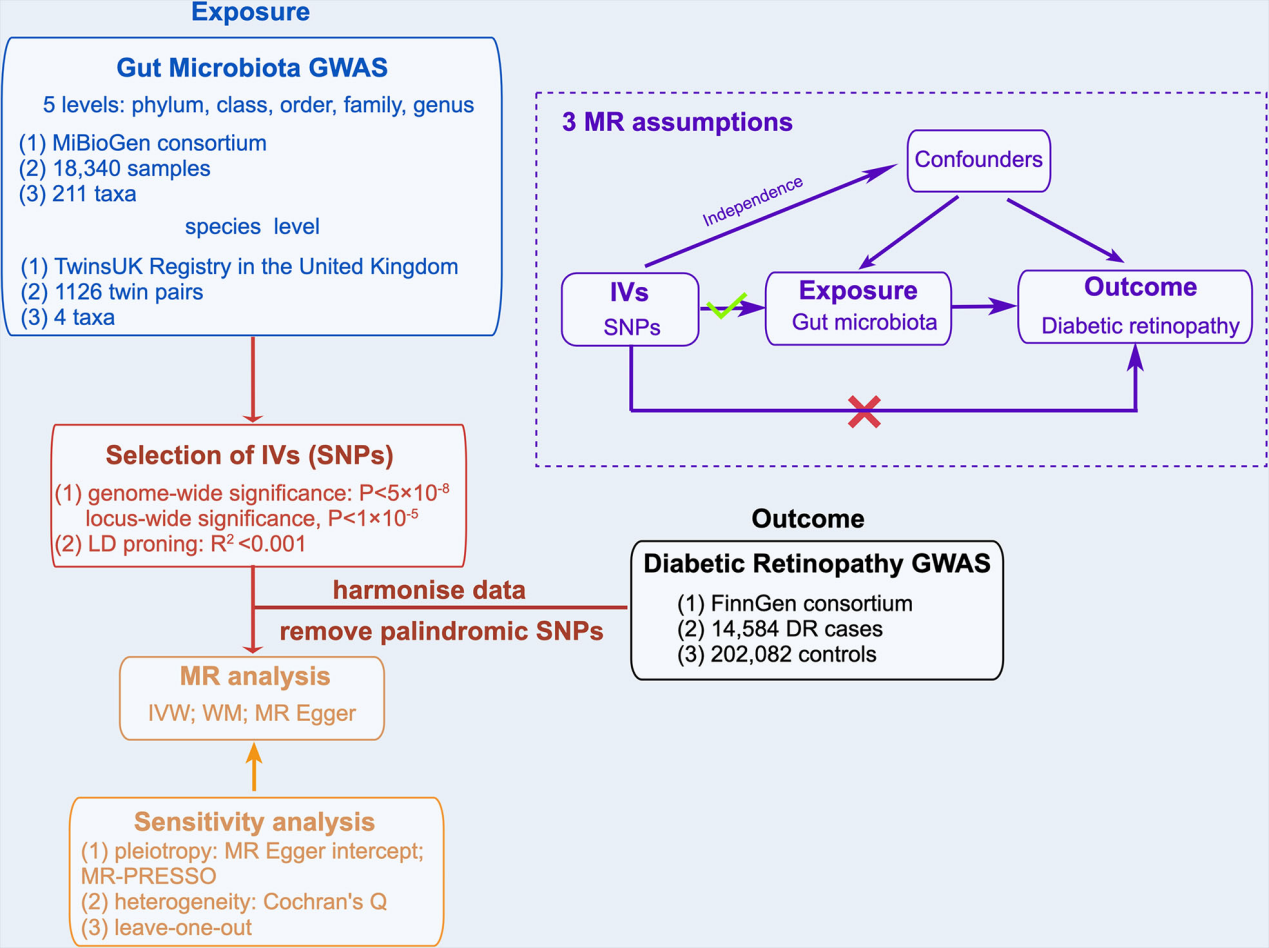
Overview of MR analyses process and major assumptions.

A two-sample MR analysis was used to evaluate the causal relationships between GM taxa and DR. Summary-level data from the genome-wide relationship studies (GWASs) were obtained for GM and DR. **Figure 1** shows the flowchart of the MR study between GM taxa and DR. Additionally, to obtain reliable results, MR analysis satisfied the following 3 assumptions (16) (**Figure 1**): (1) The instrumental variables (IVs) eventually incorporated for use must be closely related to GM taxa; (2) The included IVs and confounders (affecting GM taxa and DR) were independent of each other; (3) No horizontal pleiotropy: IVs affected DR only through GM taxa. Meanwhile, our findings were reported in adherence to the MR-STROBE guidance (17).

### 2.2 Ethics statement

The summary-level data used in this study are de-identified public data and are available for download. Each GWAS involved in this study was ethically approved by the respective institutions.

### 2.3 Exposure sources of GM taxa

Based on the MiBioGen consortium, Kurilshikov et al. (18) obtained 16S rRNA gene sequencing profiles and genotyping data from 18,340 samples to investigate the relationship between genetic variation and GM. All the subjects of the MiBioGen consortium were of European ethnicity from 25 cohorts in 11 countries. The GWAS study eventually yielded 122,110 variant sites from 211 taxa (from genus to phylum level) by analyzing the GM taxa variation across different populations. We extracted IVs of GM taxa at 5 levels from this large-scale GWAS. In addition, considering that Kurilshikov et al. did not analyze at the species level, we extracted IVs of GM taxa at the species level from the GWAS study of the TwinsUK Registry (19). The 16S rRNA sequencing data of Goodrich et al. (19) was obtained from 1126 twin pairs (average age, 59 years), and finally defined 4 eligible species. More details on the GM data can be found in the original articles (18, 19).

To ensure data robustness and the accuracy of results, the SNPs were quality checked to obtain compliant IVs: (1) SNPs associated with GM taxa reached genome-wide significance threshold (*P*<5×10^-8^). Since the number of eligible IVs (*P*<5×10^-8^) was extremely small, a relatively more comprehensive threshold (*P*<1×10^-5^) (20, 21) was selected to obtain a more comprehensive result. (2) To meet the MR assumptions, we performed a linkage disequilibrium (LD) analysis (R^2^ <0.001, clumping distance = 10,000kb) based on European-based 1,000 Genome Projects and removed the SNPs that did not meet the requirements. (3) The palindromic SNPs were removed to prevent the effect of alleles on the outcome of causality between GM taxa and DR.

### 2.4 Outcome sources of DR

The DR GWAS summary statistics were extracted from the FinnGen research project (https://r5.finngen.fi/). This GWAS analyzed 16,962,023 variables from 218,792 subjects through SAIGE (https://github.com/weizhouUMICH/SAIGE). After adjusting for age, sex, genetic relatedness, genotyping batch, and first 10 principal components, 14,584 DR cases and 202082 controls were used for DR analysis.

### 2.5 Statistical analysis

All statistical analyses were performed using the R software (Version 4.1.1). The R package “TwoSampleMR” was used to perform MR analysis of the causal relationship between GM taxa and DR. *P*<0.05 was considered the statistical significance of evidence for potential causal effect (22, 23).

#### 2.5.1 MR analysis

The Wald ratio (WR) method was utilized to examine the effect of individual IVs on the causal estimates. In the absence of horizontal pleiotropy, the inverse variance weighted (IVW) test was used as the primary method for calculating the causal effect values to obtain unbiased estimates. A fixed/random effects model was selected for the IVW test based on the presence or absence of heterogeneity. OR and 95% confidence interval (CI) showed the effect size. The weighted median (WM) method (24) and the MR-Egger test (25) were utilized as additional methods for MR analysis. WM results were used as the significant causal effect values if the number of SNPs with heterogeneity exceeded 50%. MR Egger’s results remained valid if SNPs with pleiotropy were above 50%.

#### 2.5.2 Sensitivity analysis

Cochrane’s Q test was applied to test for heterogeneity. IVs with *P*<0.05 were considered heterogeneous. The intercept of MR Egger regression assessed the presence of potential pleiotropy in IVs. Horizontal pleiotropy was deemed to be non-existent if *P >*0.05. To ensure the accuracy of results for GM taxa causally related to DR (based on IVW results), the multipotency was further analyzed using the MR-Pleiotropy RESidual Sum and Outlier (MR-PRESSO) test (R package “MR-PRESSO”), and possible outliers were removed. Additionally, the leave-one-out method (23) was used to validate data robustness further. A reverse MR analysis was not performed due to the lack of SNPs (related to DR) fitting the hypothesis of the MR study.

## 3 Results

### 3.1 Selection of IVs related to GM

After quality control steps by LD effects and palindromic, 2,249 SNPs (based on the MiBioGen consortium) were identified as IVs associated with 211 bacterial taxa for DR (*P*<1×10^-5^). These taxa comprised 9 phylum (102 SNPs), 16 class (178 SNPs), 20 order (215 SNPs), 35 family (382 SNPs) and 131 genera (1372 SNPs). Based on the TwinsUK Registry, 15 SNPs were identified as IVs associated with 4 species. Each SNP revealed adequate validity (ranged between 14.59 and 88.43, all F>10) (**Table 1**). **Supplementary Table 1** details the major information of the IVs.

**Table 1:** Selection of IVs after quality control.

Taxonomies	Taxa	NSNP	Palindromic	IVs
Phylum	9	117	15	102
Class	16	213	35	178
Order	20	263	48	215
Family	35	455	73	382
Genus	131	1626	254	1372
Total	211	2674	425	2249

When the GM was considered as a whole, only 15 SNPs passed quality control to be applied as IVs (*P*<5×10^-8^). Each SNP revealed adequate validity (ranged between 29.81 and 85.38, all F>10). 24 SNPs were identified as IVs associated with 211 bacterial taxa for DR (*P*<5×10^-8^). These taxa comprised 1 phylum (1 SNP), 1 class (1 SNP), 2 order (3 SNPs), 5 family (6 SNPs) and 12 genera (13 SNPs). Based on the TwinsUK Registry, 3 SNPs were identified as IVs associated with 2 species. Each SNP revealed adequate validity (ranged between 29.35 and 88.43, all F>10). **Supplementary Table 2** details the major information of the IVs.

### 3.2 Results of MR analysis (locus-wide significance, *P*<1×10^-5^)

#### 3.2.1 MR results at 5 levels from the MiBioGen consortium

**Figure 2:**
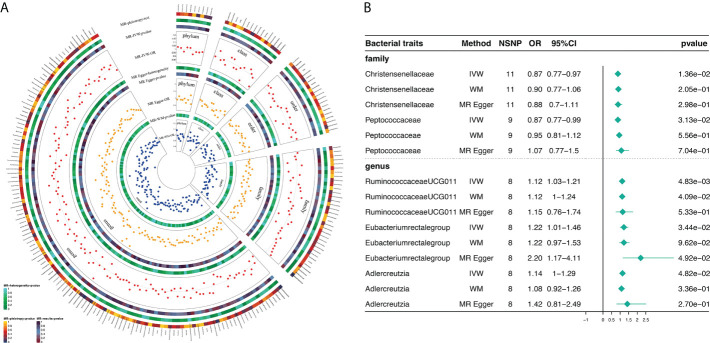
Causal analysis of GM and DR (locus-wide significance, *P*<1×10^-5^). **(A)** All results of MR analysis and sensitivity analysis between GM and DR; **(B)** MR results of GM taxa with a causal relationship to DR.

Based on MR analysis, **Figure 2A** shows the relationship between 211 bacterial taxa and DR; comprehensive results are shown in **Supplementary Table 3**. Among the MR results, we found a genetically predicted relative abundance of 2 families and 3 genera. As for the biological family classifications, the IVW results demonstrated a protective effect of the host-genetic-driven increase in *Christensenellaceae* (OR, 0.87; 95% CI, 0.77–0.97; *P* = 1.36×10^-2^) and *Peptococcaceae* (OR, 0.88; 95% CI, 0.77–0.99; *P* = 3.13×10^-2^) on the risk of DR (**Figures 2A, B**). As for the genus, the MR estimates of IVW indicated that *Ruminococcaceae*_*UCG*_*011* (OR, 1.12; 95% CI, 1.03–1.21; *P* = 4.83×10^-3^), *Eubacterium*_*rectale*_group (OR, 1.22; 95% CI, 1.01-1.46; *P* = 3.44×10^-2^) and *Adlercreutzia* (OR, 1.14; 95% CI, 1.00-1.29; *P* = 4.82×10^-2^) were risk factors for DR (**Figures 2A, B**). Also, the relationships between *Ruminococcaceae*_*UCG*_*011* and DR (OR, 1.12; 95% CI, 1.00–1.24; *P* = 4.09×10^-2^) were supported by WM (**Figure 2B**). Nevertheless, the other 4 GM taxa did not show a relationship with DR by WM (**Figure 2B**).

#### 3.2.2 MR results at the species level from the TwinsUK Registry

**Table 2** shows the relationship between 4 GM taxa at the species level and DR. Among the MR results at the species level, *Eggerthella lenta* (IVW : OR, 1.00; 95% CI, 1.00–1.00; *P* = 0.98), *Akkermansia muciniphila* (IVW: OR, 0.99; 95% CI, 0.95–1.04; *P* = 0.73), *Faecalibacterium prausnitzii* (IVW: OR, 0.99; 95% CI, 0.97–1.01; *P* = 0.41; WM: OR, 1.00; 95% CI, 0.97–1.03; *P* = 0.91; MR Egger: OR, 1.01; 95% CI, 0.98–1.04; *P* = 0.58) and *Veillonella dispar* (WR: OR, 0.99; 95% CI, 0.99–1.00; *P* = 0.71) have no effect on the risk of DR (**Table 2**).

### 3.3 Sensitivity analysis

**Supplementary Table 4** shows the pleiotropy and heterogeneity test results for all bacterial taxa (from species to phylum level), respectively. In sensitivity analysis, we confirmed the effects of accurate MR results in 2 families and 3 genera on DR. No horizontal pleiotropy was observed in *Christensenellaceae* (*P*=0.89), *Peptococcaceae* (*P*=0.24), *Ruminococcaceae*_*UCG*_011 (*P*=0.90), *Eubacterium*_*rectale*_group (*P*=0.10) and *Adlercreutzia* (*P*=0.46) for DR (**Table 2**). Meanwhile, no heterogeneity was found in *Christensenellaceae* (IVW: *P*=0.47; MR Egger: *P*=0.38), *Peptococcaceae* (IVW: *P*=0.38; MR Egger: *P*=0.43), *Ruminococcaceae*_*UCG*_*011* (IVW: *P*=0.46; MR Egger: *P*=0.35), *Eubacterium*_*rectale*_group (IVW: *P*=0.35; MR Egger: *P*=0.66) and *Adlercreutzia* (IVW: *P*=0.81; MR Egger: *P*=0.80) for DR (**Table 2**).

**Table 2:** MR results between GM at the species level and DR.

GM	Method	IVs	OR	95%CI	*P*
locus-wide significance, *P*<1×10^-5^					
*E. lenta*	IVW	2	1.00	1.00-1.00	0.98
*A. muciniphila*	IVW	2	0.99	0.95-1.04	0.73
*F. prausnitzii*	IVW	9	0.99	0.97-1.01	0.41
*F. prausnitzii*	WM	9	1.00	0.97-1.03	0.91
*F. prausnitzii*	MR Egger	9	1.01	0.98-1.04	0.58
*V. dispar*	WR	1	1.00	0.99-1.01	0.71
genome-wide statistical significance, *P*<5×10^-8^					
*E. lenta*	WR	1	1.00	1.00-1.00	0.33
*F. prausnitzii*	IVW	2	1.00	0.96-1.03	0.92

MR, Mendelian randomization; IVW, Inverse variance weighted; WM, Weighted median; WR, Wald ratio; E. lenta, Eggerthella lenta; A. muciniphila, Akkermansia muciniphila; F. prausnitzii, Faecalibacterium prausnitzii; V. dispar, Veillonella dispar.

The statistically significant MR results were further verified using MR- PRESSO to ensure the accuracy of MR Egger regression; the absence of horizontal pleiotropy was confirmed in family*-Christensenellaceae* (*P*=0.523), family*-Peptococcaceae* (*P*=0.439), *genus-Ruminococcaceae*_*UCG*_*011* (*P*=0.520), genus*-Eubacterium*_*rectale*_group (*P*=0.088) and genus*-Adlercreutzia* (*P*=0.960) (**Table 2**). Moreover, the leave-one-out results further validated data robustness (**Supplementary Figures 1A–1E**). In the absence of heterogeneity and pleiotropy, the results of IVW were reliable. Therefore, *Christensenellaceae*, *Peptococcaceae*, *Ruminococcaceae*_*UCG*_*011*, *Eubacterium*_*rectale*_group, and *Adlercreutzia* were causally related to DR.

### 3.4 Results of MR analysis (genome-wide statistical significance, *P*<5×10^-8^)

MR results of GM as a whole and DR did not reveal a significant causal relationship with DR (IVW: OR= 1.01,95%CI, 0.94-1.09, *P*=0.70; WM: OR= 1.00,95%CI, 0.91-1.10, *P*=0.0.96; MR Egger: OR= 0.98,95%CI, 0.77-1.26, *P*=0.0.88) (**Table 3**). The heterogeneity analysis results (IVW: *P*=0.64; MR Egger: *P* =0.56) and pleiotropy analysis (MR Egger: *P*=0.79; MR-PRESSO: *P*=0.64) confirmed the accuracy of the results (**Table 4**). Meanwhile, the leave-one-out results further validated data robustness (**Supplementary Figure 1F**).

**Table 3:** Sensitivity analysis between GM and DR.

Taxonomies	GM	Method	Q	*P*	Intercept	*P*	MR-PRESSO
family	*Christensenellacea*	IVW	9.69	0.47	-1.44×10^-3^	0.89	0.523
		MR Egger	9.67	0.38			
family	*Peptococcaceae*	IVW	8.62	0.38	-0.02	0.24	0.439
		MR Egger	6.96	0.43			
genus	*Ruminococcaceae_UCG_011*	IVW	6.74	0.46	-3.67×10^-3^	0.90	0.520
		MR Egger	6.72	0.35			
genus	*Eubacterium_rectale_group*	IVW	7.84	0.35	-0.04	0.10	0.088
		MR Egger	4.15	0.66			
genus	*Adlercreutzia*	IVW	3.72	0.81	-0.02	0.46	0.960
		MR Egger	3.09	0.80			

MR, Mendelian randomization; IVW, Inverse variance weighted; WM, Weighted median.

**Table 4:** MR results between GM and DR (*P*<5×10^-8^).

GM	Method	IVs	OR	95%CI	*P*	Q	Q-*P*	Intercept	*P*	MR-PRESSO
Total	IVW	15	1.01	0.94-1.09	0.70	11.63	0.64	3.78×10^-3^	0.79	0.64
Total	WM	15	1.00	0.91-1.10	0.96					
Total	MR Egger	15	0.98	0.77-1.26	0.88	11.55	0.56			

MR, Mendelian randomization; IVW, Inverse variance weighted; WM, Weighted median.

**Figure 3:**
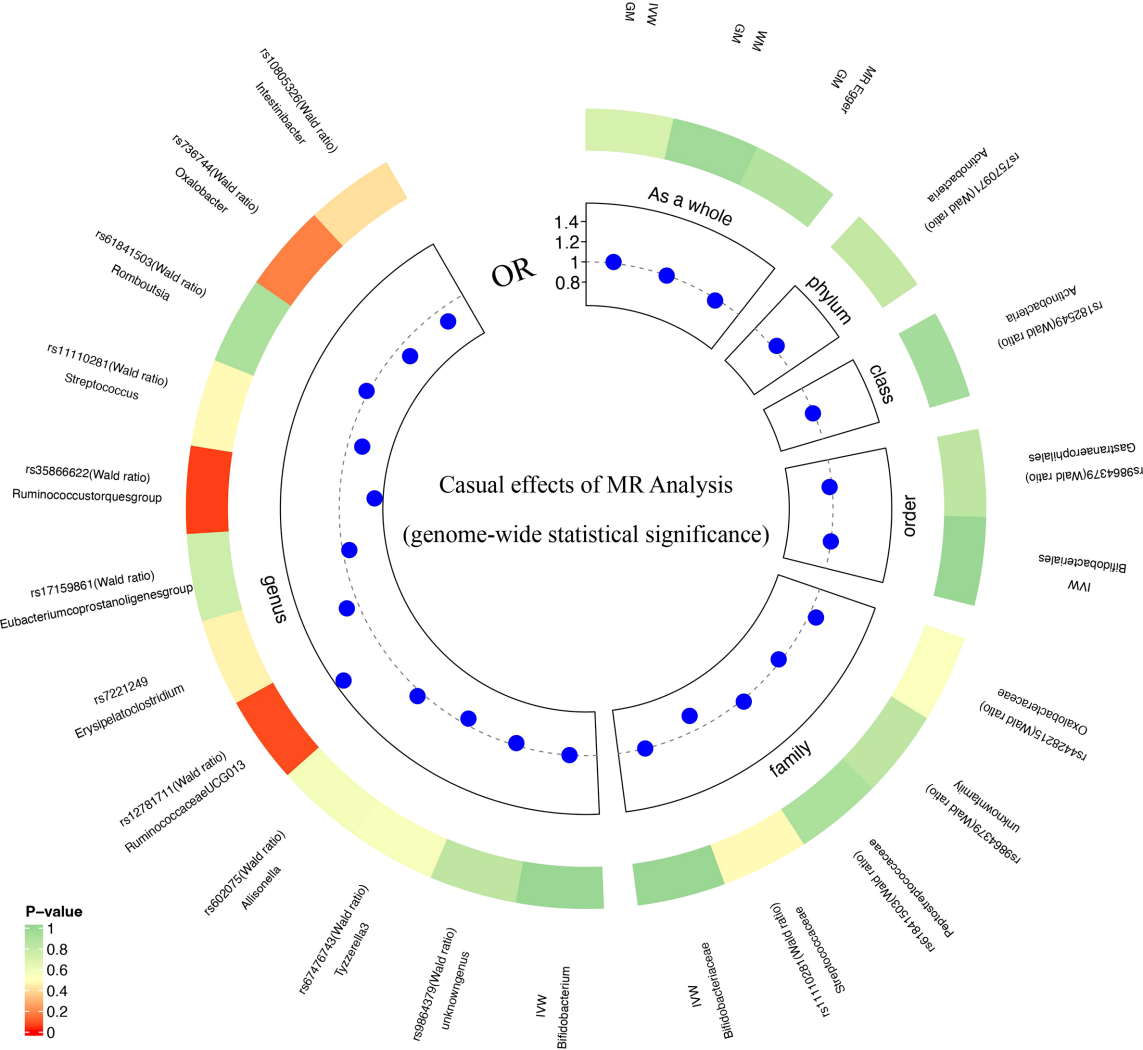
MR analysis results of GM and DR (genome-wide statistical significance, *P*<5×10^-8^).

Due to the limited number of IVs that met the requirements, none of the MR results for individual classifications of bacterial taxa at 5 levels revealed a significant causal relationship with DR (*P*>0.05) (**Figure 3**; **Supplementary Table 5**). At the species level, *Lenta* and *Prausnitzii* did not show the causal relationship with DR (*P*>0.05) (**Table 2**). Regretfully, the number of eligible IVs was too small to allow for sensitivity analysis.

## 4 Discussion

Research progress in the “gut-retina” axis has been hindered by several confounding factors (e.g., dietary habits); hence difficult to cross-sectionally examine the relationship between GM and DR. Using MR analysis, we assessed the potential causal relationship between GM taxa and DR from a host genetic perspective and confirmed the effect in modifying susceptibility to DR.At the phylum, class, order and species levels, no association between GM taxa and DR risk was found. However, 2 family taxa were linked to a low DR risk and 3 genus taxa were associated with a high risk of DR, which may promote the discovery of novel biomarkers in future DR experiments. Meanwhile, our findings provide novel ideas for future DR prevention and therapeutic approaches: targeted regulation of dysbiosis of specific GM taxa to prevent and treat DR.

Over 90% of GM comprises 4 bacterial phyla, including *Firmicutes* and *Actinobacteria* (26). When GM dysbiosis occurs, it causes metabolic, immune, and neurological-related disorders (14, 27, 28). Nonetheless, the role of GM dysbiosis in the pathogenesis of DR remains elusive. Larsen et al. (29) discovered a significantly lower proportion of phylum *Firmicutes* from the fecal bacteria of 18 diabetic patients compared to the controls. As one of the most prevalent complications of diabetes, DR has similar relationships with diabetes. Beli et al. (12) revealed that IF minimizes the risk of DR by reducing immune infiltration of retinal capillaries through increased levels of *Firmicutes* in diabetic mice. At the same time, Beli et al. (12) suggested that the GM intervention will be a novel approach for DR prevention and treatment.

Interestingly, 4 GM taxa, including *Christensenellaceae*, *Peptococcaceae Ruminococcaceae*_UCG_011, and *Eubacterium*_*rectale*_group of our results belong to phylum *Firmicutes*; which are consistent with the findings of Beli et al. Nonetheless, despite considering the GM taxa as a whole or at the phylum level, we did not discover the causal relationship between GM and DR. Ye et al. (30) compared 45 patients with proliferative DR and 90 diabetes patients. Consequently, no significant difference in GM was found between the two groups at the phylum level, which is in line with our findings. The difference in results was attributed to several reasons. First, this work and Ye et al. were performed on human samples, whereas Beli et al. were mice models, which inevitably caused differences in GM abundance. Furthermore, similar to our consideration of GM as a whole, GM at the phylum level covered too many taxa. The interplay of effects between the different taxa in the refinement (e.g., level of family and genus) could have affected the observation of the impact.

Fernandes et al. (31) revealed that diabetes-associated GM disrupts the inflammation level, influencing DR progression. The abundance of *Christensenellaceae* was higher in the healthy population, and its abundance was inversely correlated with inflammation (22). Although no studies on *Christensenellaceae* in eye disease have found an effect on inflammation, studies on *Christensenellaceae* in inflammatory bowel disease (IBD) have laterally confirmed a possible effect on the retina *via* inflammation. Similar to DR and IBD, Rosacea is considered an inflammatory disease. Nam et al. (32) also found a lower abundance of *Peptococcaceae* in rosacea patients, which follows a similar trend to our results. Therefore, we speculate that *Christensenellaceae* and *Peptococcaceae* might reduce inflammatory damage to the retina through the “intestinal-retinal axis,” thereby affecting disease progression in DR.

Unlike other GM and eye disease studies, we further identified 3 taxa at the genus level, increasing the DR risk. However, there is a lack of ophthalmic research evidence to confirm the specific mechanism by which these 3 GM taxa increase DR risk. By inhibiting CD 83, Islam et al. (33) discovered that *Eubacterium*_*rectale*_group could induce systemic inflammation and activate dendritic cells. Also, Wang et al. (34) confirmed the proinflammatory effect of *Eubacterium*_*rectale*_group. Therefore, we speculate that the *Eubacterium*_*rectale*_group exacerbates retinal vascular damage *via* systemic inflammation, thereby increasing the risk of DR. Yuan et al. (35) discovered that *Adlercreutzia* is associated with androgen levels. Interestingly, increased androgen levels were linked to DR progression (36). For *Ruminococcaceae*_*UCG*_*011*, no studies have yet reported its association with disease. For the first time, our findings confirm the pathogenic potential of *Ruminococcaceae*_*UCG*_*011* in humans, implying that this taxon could be a novel biomarker. Nonetheless, its specific should be further explored.

This study has several advantages. First, the current relationship between GM and eye disease has primarily focused on the family classification level. Our analysis further provides refinement of the GM taxa, analyzing the causal effect of each taxon on DR from the genus to the phylum level. This provides a theoretical basis for the follow-up mechanisms of specific strains of bacteria on DR and facilitates the discovery of new biomarkers. Secondly, the latest large GWAS allows genetic data to be obtained from large sample populations and analyzed, hence cementing the reliability of findings compared to small randomized controlled studies. On the other hand, the MR analysis prevents confusion and provides a new approach to investigating the “gut-retina axis” mechanisms.

This work also has compelling limitations. Similar to other GM-related MR studies, there is still no guarantee of weak instrumental bias despite satisfying the MR assumptions (IVs are closely correlated with GM taxa). Secondly, although the symptoms of DR were confined to the eye with subtle or even no effects on GM, we were unable to identify a possible mutual causal relationship between GM and DR due to the lack of an adequate number of IVs for reverse MR analysis. Thirdly, since the GWAS included subjects only of European descent, the findings of this study may not be generalizable to other ethnic groups. Fourth, multiple statistical corrections are too strict and conservative and may ignore GM taxa with a possible causal relationship with DR. Therefore, we did not consider the multiple testing results given the biological plausibility. In addition, this is the first time that we have used MR study to analyze the relationship between GM taxa and DR risk at the species level, but we have not found a causal relationship with DR at the species level. Considering that the data source of species level is different from the other five levels, and the GWAS sample size of Goodrich et al. (19) is less than that of Kurilshikov et al. (18), the number of IVs that could ultimately be selected remained limited. Therefore, this discovery at the species level is only a preliminary exploration. In future research, we will expand the sample as much as possible to explore the relationship between GM taxa and DR at the species level, to provide more theoretical support for the mechanism research of the “gut-retina” axis.

In conclusion, we confirmed a causal relationship between DR and GM taxa, including *Christensenellaceae*, *Peptococcaceae*, *Ruminococcaceae_UCG_011*, *Eubacterium*_*rectale*_group, and *Adlercreutzia*. These strains may become novel biomarkers and provide insights for the treatment and prevention of DR.

## Data availability statement

The original contributions presented in the study are included in the article/**Supplementary Material**. Further inquiries can be directed to the corresponding author.

## Ethics statement

Ethical review and approval was not required for the study on human participants in accordance with the local legislation and institutional requirements. Written informed consent for participation was not required for this study in accordance with the national legislation and the institutional requirements.

## Author contributions

KL and ZY designed the study. KL, JZ, and ZY analyzed the data and drew the figures. All authors critically revised the manuscript. All authors read and approved the final manuscript.

## Funding

This work was supported by grants from the National Natural Science Foundation of China (81860175), Science and Technology Innovation Base Construction - Clinical Medicine Research Centre Project (20221ZDG02012) and the Jiangxi Natural Science Foundation (20202BAB206035) to ZY; the postgraduates innovation special fund project of Jiangxi province (YC2022—B051) and a grant from the Talent Development project of the Affiliated Eye Hospital of Nanchang University (No. 2022X05) to KL. The funders had no role in the study design, data collection, data analysis, interpretation, or writing of the report.

## Acknowledgments

We want to acknowledge the participants and investigators of FinnGen study (https://finngen.gitbook.io/documentation/) and MiBioGen consortium for sharing the genetic data. We thank Figdraw (www.figdraw.com) for expert assistance in **Figure 1**.

## Conflict of interest

The authors declare that the research was conducted in the absence of any commercial or financial relationships that could be construed as a potential conflict of interest.

## Publisher’s note

All claims expressed in this article are solely those of the authors and do not necessarily represent those of their affiliated organizations, or those of the publisher, the editors and the reviewers. Any product that may be evaluated in this article, or claim that may be made by its manufacturer, is not guaranteed or endorsed by the publisher.

## Supplementary material

Supplementary Figure 1 The leave-one-out results of GM and DR (locus-wide significance, *P*<1×10^-5^). **A.** The leave-one-out analysis for family-Christensenellaceae on DR (locus-wide significance, *P*<1×10^-5^). **B.** The leave-one-out analysis for family-Peptococcaceae on DR (locus-wide significance, *P*<1×10^-5^). **C.** The leave-one-out analysis for Genus-Ruminococcaceae_UCG_011 on DR (locus-wide significance, *P*<1×10^-5^). **D.** The leave-one-out analysis for genus-Eubacterium_rectale_group on DR (locus-wide significance, *P*<1×10^-5^). **E.** The leave-one-out analysis for enus-Adlercreutzia on DR (locus-wide significance, *P*<1×10^-5^). **F.** The leave-one-out analysis for GM as a whole on DR (genome-wide statistical significance, *P*<5×10^-8^).
