# Supplementary figures and images for: Causal effects of gut microbiota on diabetic retinopathy: A Mendelian randomization study
Source: Front Immunol. 2022 Sep 8;13:930318. doi: 10.3389/fimmu.2022.930318 (PMC9496187; doi:10.3389/fimmu.2022.930318)

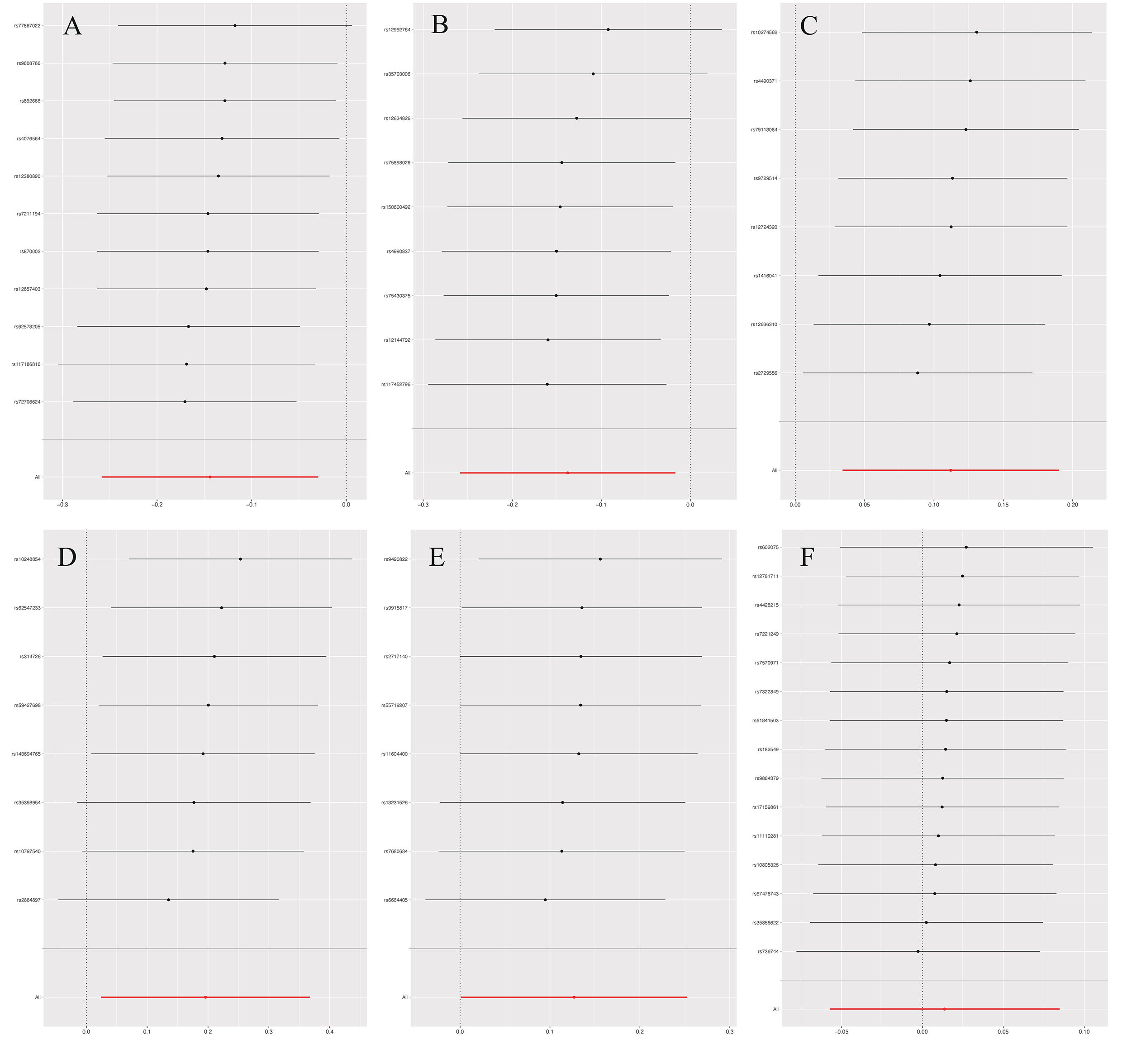

Supplement: Supplementary Figure 1 — The leave-one-out results of GM and DR (locus-wide significance, P<1×10^-5^). A. The leave-one-out analysis for family-Christensenellaceae on DR (locus-wide significance, P<1×10^-5^). B. The leave-one-out analysis for family-Peptococcaceae on DR (locus-wide significance, P<1×10^-5^). C. The leave-one-out analysis for Genus-Ruminococcaceae_UCG_011 on DR (locus-wide significance, P<1×10^-5^). D. The leave-one-out analysis for genus-Eubacterium_rectale_group on DR (locus-wide significance, P<1×10^-5^). E. The leave-one-out analysis for enus-Adlercreutzia on DR (locus-wide significance, P<1×10^-5^). F. The leave-one-out analysis for GM as a whole on DR (genome-wide statistical significance, P<5×10^-8^). [file Image_1.tif]
